# Correction to “Quality of vitamin K antagonist treatment during the last year of life”

**DOI:** 10.1002/hem3.70173

**Published:** 2025-07-28

Visser C, Kempers EK, Goedegebuur J, et al. Quality of vitamin K antagonist treatment during the last year of life. *HemaSphere*. 2025;9(5):e70135. https://doi.org/10.1002/hem3.70135.

In the author listing of the manuscript, the name of an author was incorrectly listed as Jamilla Goedgebuur. The correct name is Jamilla Goedegebuur.

The original publication has been corrected. We apologize for this error.

